# Efficacy of multiplex PCR procedure for Iranian Streptococcus pneumoniae isolates

**Published:** 2014

**Authors:** Massoud Hajia, Mohamad Rahbar, Marjan Rahnami Farzami, Alireza Dolatyar, Mohsen Imani, Roghieh Saburian, Mohamad Farzanehkhah

**Affiliations:** 1 Health Reference Laboratories Research Center, Ministry of Health & Medical Education, Tehran, Iran.

**Keywords:** *S. pneumoniae*, Multiplex PCR, Identification

## Abstract

***Background: ***Identifying regional types and evaluating the frequency of pneumococcal strains has become increasingly important especially in vaccination. The purpose of this study was the identification and frequency determination of our regional serotype and evaluation of the performance of recent type specific multiplex PCR for the diagnosis of streptococcus pneumonia serotypes.

***Methods:*** All isolated *S. pneumonia* from suspected patients in Tehran and Isfahan Hospitals from June to December of 2012 were evaluated. All specimens and their serotypes were identified through a conventional method and specific antisera. Serotype specific multiplex PCR was applied and ran in seven reactions consisting of 34 *S. pneumonia* primer pairs plus a primer pair as an internal control for this purpose.

***Results:*** Overall, 14 genotype specific serotypes (including two subtypes for 19 and 23) were detected and had identical results with stereotyping method except for serotype 28 and one of the identified serotype 23. The serotypes 19, 6 and 23 were dominant with the frequency of 51.8%. A cross reactivity was also observed between genotypes 1 and 9A/9V.

***Conclusion: ***Applied multiplex PCR format can be suitable and cost effective tool for identification of *S. pneumonia* serotypes.

The considerable rate of pneumococcal meningitis and bacteremia and also the spread of high-level resistance to penicillin have focused attention towards disease prevention, with re-examination of the role and potential impact of more widespread vaccination (1-3). Identifying and frequency detecting of pneumococcal serotypes are important for surveillance programs and for evaluating the efficacy of vaccination on pneumococcal infections. *S. pneumoniae* can produce over 90 immunologically distinct capsules that differ in chemical structure (4). More importantly, despite the large variety of capsular types, only a small fraction of these causes most cases of invasive diseases (5). Accurate assessment of the invasive serotype distribution could be of great value in the evaluation and formulation of pneumococcal vaccines. Various molecular typing methods for pneumococci have been described such as ribotyping, DNA probe, pulsed-field gel electrophoresis of large DNA restriction fragments, and multiplex PCR (3-7). Serotyping was frequently reported using the sequential multiplex PCR method (SM-PCR) described by Pai et al. in 2006 (8, 9). They have adapted the multiplex format to their regional algorithm observed in Burkina Faso and Togo, in which 29 different serotypes were grouped into seven PCRs.

It seems the development of PCR-based stereotyping systems is a strong alternative way and has the suitable potential role to overcome some of the difficulties associated with current serologic testing conditions. Recently, the CDC has introduced three multiplex PCR protocols for the different parts of the world to identify pneumococcal serotypes (9). Therefore, reliable typing system is important to detect dominant strains. The aim of this study was to compare pneumococcal typing using multiplex PCR with confirmed isolated serotypes, and also detecting the frequency of invasive pneumococcal serotypes.

## Methods

From June to December 2012, all isolated *S. pneumonia* from suspected patients were evaluated. Overall, 29 patients with *Streptococcus pneumonia* were isolated and identified by conventional method from patients with suspect to have pneumococcus pneumonia and meningitis at Milad, Bahrami and Shariati Hospitals in Tehran and Alzahra, Amin and Mahdyieh Hospitals in Isfahan. Conventional tests and PCR method were performed by two separate laboratory staff, and dissimilar results were repeated to ensure of the accuracy of final outcome.

Received specimens were CSF, eye discharge, pleural fluid and trachea aspirates. The mentioned samples were cultured on blood agar. Suspected colonies were identified by hemolysis pattern, sensitivity to optochin and bile solubility. All isolates were cultured in TSB media to preserve them which contained 10 % glycerol and were maintained in the temperature of -70 Celsius degree. Then, all pneumococcal isolates were typed by Mast Assure^TM^ Streptococcus pneumonia typing antisera (MAST GROUP LTD. Merseysid, U.K.). The kit consists of 8 polyvalant antisera and covers 39 of the following types: 1, 2, 3, 4, 5, 6, 7, 8, 10 , 11, 12, 14, 15, 16, 17, 18, 19, 20, 21, 22, 23, 24,25, 27, 28, 29, 31, 31, 33, 34, 35, 36, 38, 39, 41, and 47.

**PCR protocols: **Microbial suspension was prepared for each isolated specimen and extracted using of QIAamp DNA Mini Kit (QIAGEN GmbH, Hilden, Germany). The purity of all the extracted specimens was determined by measuring the optical density at the wavelengths of 260 and 280 nm. Thirty four type specific primer pairs were chose from CDC designed to target the following serotypes: 1, 2, 3, 4, 5, 6A/6B/6C, 6C, 7C(7B/40), 7F/7A, 8, 9N/9L, 9V/9A, 10A, 11A/11D, 12F/(12A/44/46), 14, 15A/15F, 15B/15C, 16F, 17F, 18/(18A/18B/18C/18F), 19A, 19F, 20, 22F/22A, 23A, 23F, 31, 33F/(33A/37), 34, 35B, 35F/47F, 38/25F (10, 11). Besides the primer pair targeting cpsA gene with amplification size, 160 base pairs were used as internal control (table 1). 

**Table 1:** Pneumococcal serotypes used in this study

**Product size **	**Serotype**	
189 280 362 430 566 816	14 1, 5, 4, 19A, 9V/9A	Multiplex PCR 1
250 304 384 573 826	6A/B/C 19F, 23F, 18/(18A/18B/18C), 7F/7A	Multiplex PCR 2
201 376 516 643 712	8 12F(12A/44/46), 9L/9N, 22F/22A, 23A,	Multiplex PCR 3
260 371 463 574 693	7C/7B/40, 3, 15B/C, 25F/38, 17F	Multiplex PCR 4
290 480 463 514 624 701	2, 34, 11A/11D, 20, 10A, 31,	Multiplex PCR 5
338 436 517 677 988	33F/33A/37, 15A/15F, 35F, 35B, 16F	Multiplex PCR 6
250 727 160	6A/6B/C, 6C cpsA	Multiplex PCR7

PCR was run by seven multiplex (table 1). Twenty-five μl mixtures were prepared using of 200 μM of dNTP, 3.5 mM MgCl_2_, 1x PCR buffer and the different concentrations of each primer (0.5 to 2μM). The multiplex PCR was performed on Eppendorf thermal cyclers (Mastercycler Gradient) through the following programs: 94^o^C for 4 min, then 30 cycles at 94^o^C for 45 sec, 54^o^C for 45 sec and 65^o^C for 30 sec, at the end of 2 min at 65^o^C. PCR products were analyzed on 2% agarose gel with 100 base pair ladder.

## Results

All specimens were typed by its specific antisera kit and multiplex PCR protocol as well (table 1). The comparison of the results of multiplex PCR with serotyping was confirmed in 25 tested specimens (table 2). 

**Table 2:** Comparison of the result of serotyping with the result of serotype specific multiplex PCR

	** specific antisera**	**Serotype specific multiplex **	**Explanation**
1	6	6A/6B/6C	
2	6	6A/6B/6C	
3	23	Negative	
4	19	19F	
5	19	19A	
6	6	6A/6B/6C	
7	9	9V/9A, 1	Weakly Reaction with type 1
8	19	19F	
9	4	4	
10	19	19F	
11	28	Negative	
12	17	17F	
13	23	23F	
14	23	23F	
15	3	3	
16	29,35	35	
17	1	1, 9V/9A	Strong reaction with 9V/9A
18	23	23A	
19	8	8	
20	19	19F	
21	19	19F	
22	29,35	35B	
23	6	6A/6B/6C	
24	11	11A/11D	
25	15	15A/15F	
26	9	9V/9A	
27	3	3	
28	8	8	
29	5	5	

Serotyping proved cross reaction in two samples. Specimen numbers 7 and 17 were serotypes 9 and 1 respectively, while both samples had cross reaction in multiplex PCR with each other. This was confirmed after repeating the multiplex PCR and serotyping test (picture 1). 

The rest of the two specimens had negative results, while both were type 28 and type 23 with specific antisera. Specific primer pair detecting type 28 was not included in our multiplex PCR protocol. The most prevalent observed serotypes were types 19, 23 and 6 in this study (table 3).

The serotyping results were identical in the rest specimens except for two isolates that were detected as serotypes 29 and 35 by specific antisera, while both were identified only the 29 with multiplex PCR. According to the antisera manual, 35 was a false positive result that we ignored it. Overall, sixteen specific genotyping pneumococcal serotypes were detected. Serotypes 19F and 6A/B were the most prevalent serotypes in this study 17.24%, and 13.78%, respectively and 48.25% of all the identified serotypes (table 2).

**Table 3:** Frequency of identified *S. pneumoniae* genotyping specific serotype

***S. pneumoniae *** *** Serotype***	***S. pneumonia *** **Genotypes**	**Absolute Frequency**	**Relative Frequency**
1	1	1	3.45%
3	3	2	6.89%
4	4	1	3.45%
5	5	1	3.45%
6	6A/6B	4	13.78%
8	8	2	6.89%
9	9V/9A	2	6.89%
11	11A/11D	1	3.45%
15	15B/15C	1	3.45%
17	17F	1	3.45%
19	19A	1	3.45%
19	19F	5	17.24%
23	23A	1	3.45%
23	23F	2	6.89%
28	-	1	3.45%
35	35	2	6.89%

**Figure 1:**
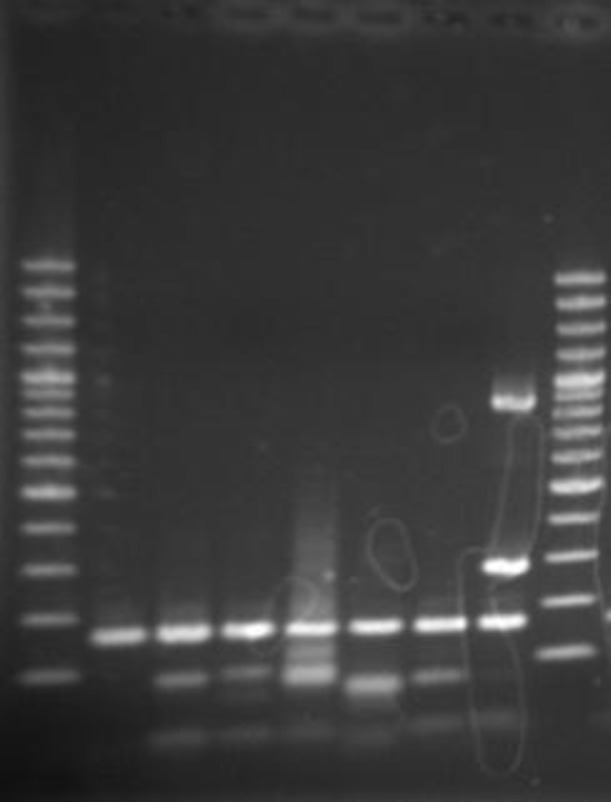
Cross, reactivity of Genotype 1 with Genotype 9

## Discussion

Serotype-specific IPD surveillance is obviously needed to continuously evaluate the applied evidence-based vaccine on invasive pneumococcal disease (IPD) and potential serotype replacement. The distribution of invasive serotypes could be affected by age, geography, and socioeconomic conditions of each specific population (1-4). Consequently, it differs in other parts of the world, although the highest worldwide nasopharyngeal colonization rates were reported from Africa (2). 

The usefulness of PCR method has already been reported for the diagnosis of *S.pneumoniae* (11). However, it was important for us to ensure of its efficacy for the detection of pneumococcal genotypes as well. The analyzed results of this study revealed all indentified serotypes by specific antisera with excellent correlation results via type specific multiplex PCR except serotype 28 that was not basically included in our multiplex PCR protocol, although one of the specimen detecting serotype 23 did not react with our specific primer pairs for 23A and 23F that could be type 23B (12). We also observed a cross reactivity between types 1, 9V/9A. Cross reactivity was previously pointed out in other reports (13-14). 

Hence, are necessary further work and analysis of primer sequences to judge about this cross reaction for future work, although it is important to include other type primer pairs at least for genotype 28 and other subspecies of type 19, since one of the specimens were identified by antisera and had negative result with pneumococcal specific genotypes 19A and 19F. In our study, the most dominant serotypes were only 19 and 6. 

 In Karimi et al.’s study they reported the prevalence pneumococcal prevalence by PCR without mentioning of the details of their procedure (15). The most observed type in their work was genotype 19 and 6 (11.86% and 10.12%, respectively), although they detected 22 pneumococcal genotypes. They tested the nasopharyngeal specimens of healthy children, while our work was based on the isolation of invasive type from patients with pneumococcal infections. However, according to these reported Iranian studies and our work, three serotypes (19, 23 and 6) seem to be the most prevalent types in our country.

 Recently, new 13-valent pneumococcal conjugate vaccine (PCV13) has been approved by FDA that covers serotypes: 1, 3, 4, 5, 6A, 6B, 7F, 9V, 14, 18C, 19A, 19F, and 23F (16). Six of our reported serotypes (8, 11A/11D, 15B/15C, 17, 28, and 35) are included in this new PVC13 vaccine. Our results show the frequency of those uncovered serotypes that was 27.58% of all the identified types in this study. There is another polysaccharide vaccine covering 23 serotypes that are commonly used (16, 17). The analyzed results confirmed 10.34% of the identified specific serotypes (28 and 35) that were not covered by this vaccine as well. In conclusion, applied multiplex PCR format can be suitable and cost effective tool for the identification of *S. pneumonia* serotypes and can be used as a screening method for detecting pneumococcal invasive serotypes. This applied protocols covering over 95% of all Iranian reported invasive types, although it is necessary to include all required serotypes considered on the multiplex format as well as those overcoming cross-reactivity between genotypes 1 and 9.

## Notes

**Funding:** The Health Reference Laboratory financially supports this project.
